# Twinning in MAPbI_3_ at room temperature uncovered through Laue neutron diffraction

**DOI:** 10.1038/s41598-020-73487-1

**Published:** 2020-10-06

**Authors:** Joachim Breternitz, Michael Tovar, Susan Schorr

**Affiliations:** 1 Department Structure and Dynamics of Energy Materials, Helmholtz-Zentrum Berlin für Materialien und Energie GmbH, Hahn-Meitner-Platz 1, 14109 Berlin, Germany; 2 Department Geosciences, Freie Universität Berlin, Malteserstr. 74-100, 12249 Berlin, Germany

**Keywords:** Solar cells, Energy, Materials science, Characterization and analytical techniques

## Abstract

The crystal structure of MAPbI_3_, the signature compound of the hybrid halide perovskites, at room temperature has been a reason for debate and confusion in the past. Part of this confusion may be due to twinning as the material bears a phase transition just above room temperature, which follows a direct group–subgroup relationship and is prone to twinning. Using neutron Laue diffraction, we illustrate the nature of twinning in the room temperature structure of MAPbI_3_ and explain its origins from a group-theoretical point-of-view.

## Introduction

Hybrid halide perovskites have caused a *nouvelle vague* in the research of photovoltaic absorber materials^1,2^. Within 10 years of their first consideration as solar absorbers, perovskite solar cells have reached over 25% efficiency^3^, close to the theoretical limit of such devices. While the rise in efficiency has provoked a huge interest in applied research—with some 4000 papers published on halide perovskites in 2019^4^, the efforts in structural elucidation have not always been commensurate. The crystal structure of methylammonium lead iodide (MAPbI_3_), for instance, has been a matter of debate and several, partially contradicting studies since its introduction into solar cells, although MAPbI_3_ clearly is the signature compound of this family of materials^5–11^. One striking discrepancy in the structural characterisation of MAPbI_3_ is the question as to whether it is ferroelectric at room temperature or not^12–20^. The generally accepted space group of MAPbI_3_ at room temperature is *I*4/*mcm*^5,7,9,11^, a centrosymmetric space group that would not allow bulk ferroelectricity or further non-linear effects. However, there are striking reports of such behaviour^16^ and we have recently discussed a deviation from the centrosymmetric crystal structure to a polar one (in space group *I*4*cm*) through the influence of the molecular cation on its surrounding iodide anions^21^.

One important factor in the structural elucidation of hybrid halide perovskites in general^20,22^ and MAPbI_3_ in particular^23–26^ is their tendency for twinning. Commonly, this twinning can be associated with the phase transition from the high-temperature phase in the cubic aristotype to the lower temperature hettotypes. Ferroelectric compounds, for instance, do normally show heavy domain twinning, as this allows the compensation of the internal electric field through the formation of oppositely polarised domains. In the case of MAPbI_3_ at room temperature, this would correspond to inversion twins in this case, as the inversion symmetry is lost between *I*4/*mcm* and *I*4*cm*. However, the observed domain structure of MAPbI_3_ exhibits further twin laws, so that twinning solely due to the loss of inversion symmetry would not explain this fully. To account for this further twinning, namely ferroelastic twinning was proposed as primary driver^23–26^. Nonetheless, it should be emphasised that ferroelasticity and ferroelectricity are not mutually exclusive but can exist in the same phase, as for instance in BiFeO_3_^27^.

At higher temperatures, MAPbI_3_ crystallises in the cubic perovskite aristotype (space group *Pm*$\bar 3$*m*). When cooling below 330 K, the crystal structure changes into a tetragonal hettotype in *I*4*cm* or *I*4/*mcm*, where the latter is the centrosymmetric and the former the polar space group choice. Since the question of centrosymmetricity is not touched in this work, we give both options in the further discussion. It needs to be mentioned that the structural differences are small, which makes the distinction between the choices even more subtle. Finally, at 160 K, the crystal structure of MAPbI_3_ transitions into an orthorhombic perovskite subgroup (space group *Pnma*)^11,28^. The high temperature phase transition is reasonably close to room temperature, and, consequently, below the typical synthesis temperature in conventional solution based processes^29^ as well as in other important techniques, such as the inverse temperature gradient method^30^. Therefore, MAPbI_3_ necessarily undergoes a phase transition when synthesised through such a way. Herein, we studied a crystal that was synthesised at room temperature and that was never heated above the cubic-tetragonal phase transition temperature. This study is seconded by an in-depth explanation of twinning in MAPbI_3_ in order to make the thematic approachable for the wider scientific community.

### Twinning in MAPbI_3_ through the cubic-to-tetragonal phase transition

In a nutshell, twinned crystals are not composed of one single orientation, but are built up from different individual or domains. These domains or individual are, however, not randomly oriented but related through point symmetry elements, which must not be element of the space group symmetry, and can be categorised through the twinning element in inversion twins (related through inversion at a twin inversion centre), rotation twins (related through rotation at a twin axis) or reflection twins (related through reflection at a twin plane)^31^. For twinning that occurs during a phase transition, the twinning element needs to be a symmetry element of the high-symmetry structure, which is lost during the phase transition. Therefore, it is crucial to understand the group-subgroup relationships through the cubic-to-tetragonal phase transition in MAPbI_3_. Prior work on twinning in the perovskite BaTiO_3_ may be taken as a first guidance^32^.

**Figure 1:**
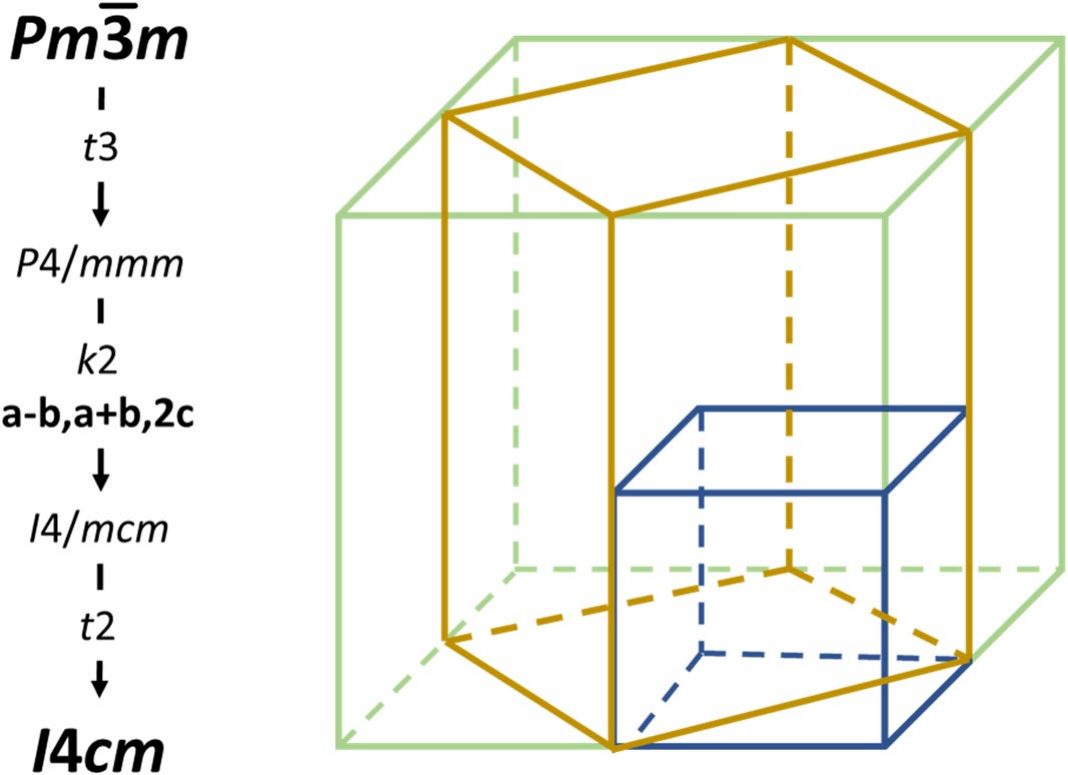
Group–subgroup relationship (left) between the cubic aristotype and the tetragonal hettotype in *I*4*cm* as the lowest possible space group type. Relationship between the unit cells of the cubic aristotype (blue), the standard setting of the tetragonal phase (gold) in *I*4*cm* (*I*4/*mcm*) and the non-standard setting *F*4*mc* (*F*4/*mmc*).

The phase transition from the cubic perovskite aristotype in *Pm*$\bar 3$*m* to the room temperature perovskite structure in *I*4*cm* (*I*4/*mcm*) follows a direct group-subgroup relationship and hence makes a second order phase transition possible. Even more importantly, the *t*3 *translationengleiche* symmetry descent from *Pm*$\bar 3$*m* to *P*4/*mmm* (Fig. 1) gives rise to the formation of triple twinning along the lost symmetry elements, i.e. the four threefold rotation axes. It is probably more illustrative to approach this from the unit cell dimensions (which are, of course, a consequence of the symmetry operations): while all three unit-cell axes are constrained to be equal in the cubic crystal system, only *a* and *b* have to fulfil this constraint in the tetragonal crystal system with *c* being independent of the other two. When transitioning from cubic to tetragonal, the choice of which of the three cubic unit-cell axes becomes the independent *c*-axis is arbitrary and hence three equivalent possibilities exist, standing perpendicular to each other.

A complication of the cubic-to-tetragonal transition in MAPbI_3_ is the symmetry descent from *P*4/*mmm* into the body-centred *klassengleiche*  subgroup *I*4*cm* (*I*4/*mcm*; Fig. 1). This transition is accompanied with a 45° rotation of the unit cell in the *ab*-plane and thus a change of the lattice constants in the manner *a*_t−I_ = √2∙*a*_c_ and *c*_t−I_ = 2∙*c*_c_ (where the index t − I signifies the tetragonal body centred structure and c the cubic one). This has as consequence that direction and plane denominations change in a rather complex manner when transforming from *Pm*$\bar 3$*m* to *I*4*cm* (*I*4/*mcm*). Therefore, we chose a non-standard setting of the tetragonal unit cell in *F*4*mc* (*F*4/*mmc*), where *a*_t−F_ = 2∙*a*_c_ and *c*_t−F_ = 2∙*c*_c_ (with t − F standing for tetragonal face centred; Fig. 1). Although this setting violates the Bravais rules of choosing the smallest possible unit cell, it has as major advantage that the directions correspond to each other in the tetragonal and the cubic lattices. Where appropriate, we will give directions and lattice planes for both settings. At room temperature, the lattice constants *a* and *c* with regard to the non-standard setting *F*4*mc* (*F*4/*mmc*) are not greatly different (*a*_t−F_ = 12.507 Å, *c*_t−F_ = 12.622 Å)^21^, which means that the diffraction spots of the different domains are close to each other to the extent that they nearly overlap completely—similar to a pseudo-merohedral twinning.

**Figure 2:**
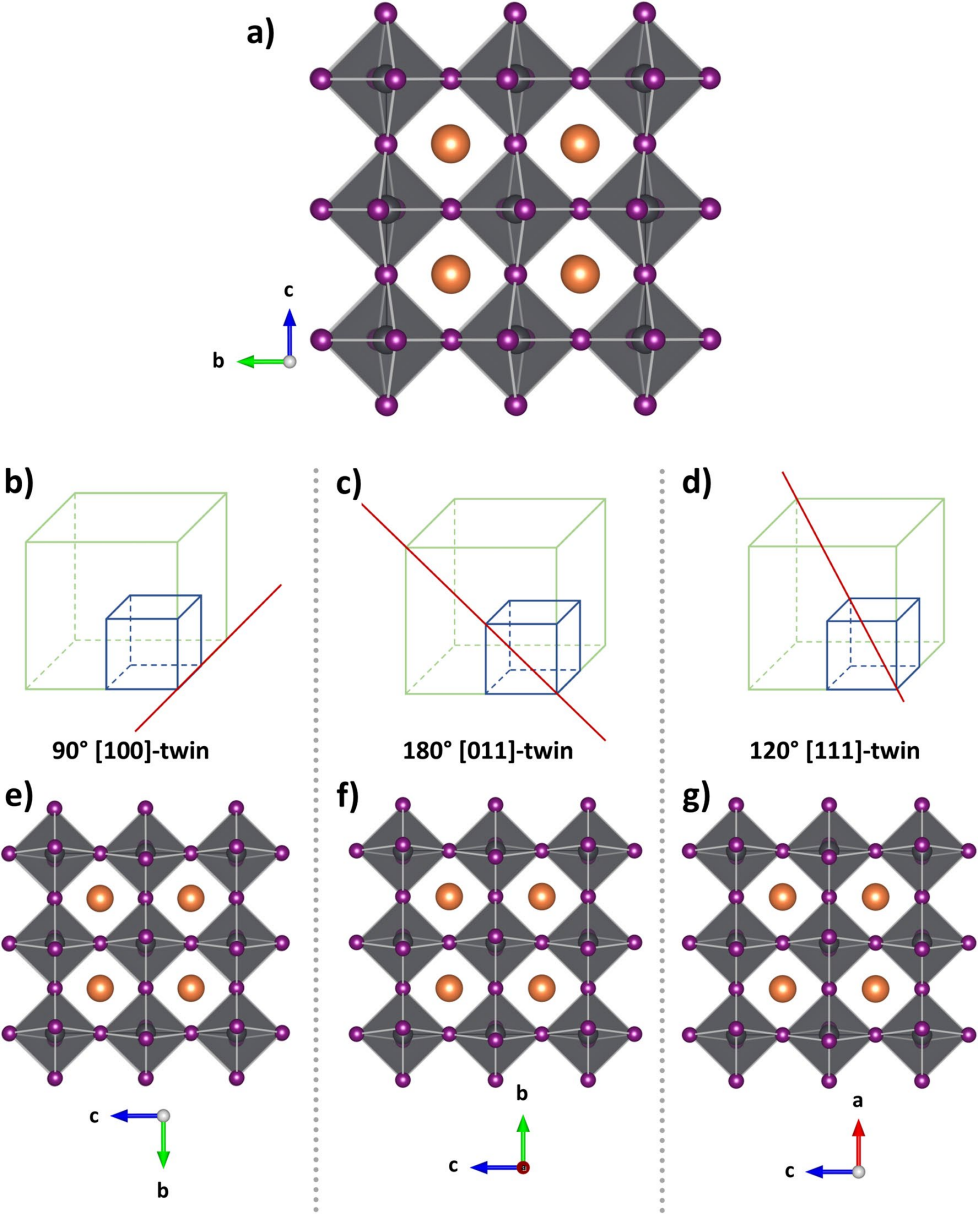
View of the crystal structure of MAPbI_3_ along the *a*-axis (with crystallographic axes according to the non-standard setting *F*4*mc* (*F*4/*mmc*)) (**a**). Representations of different rotation twin axes (red lines) in the cubic (blue) and non-standard tetragonal (green) unit cells (**b**–**d**), and the resulting crystal structures with the twinning elements acting upon the original crystal structure (**e**–**g**). The molecular cation is simplified as orange spheres for the sake of visibility.

The twinning element in the cubic-to-tetragonal descent can, essentially, be any symmetry element lost during the phase transition. Most prominently, the aforementioned three-fold axes, which are defining the cubic crystal system, can become the twinning element. However, rotation twinning through 90° rotations along the <100>_c_ axes or 180° rotation along the <101>_c_ axes (Fig. 2) are also possible as well as reflection twinning on the {011}_c_-planes^23–25^. These twin laws correspond to the respective four-fold and two-fold rotation symmetries that are lost when *c* becomes unequal from *a* and *b* in the transition from the cubic aristotype to the tetragonal structure. However, these different twin laws are only seemingly contradictory, as they all represent the same phenomenon of axis permutations, i.e. of mapping the unique *c* axis on either *a* or *b* axis (Fig. 2). The difference between the different twin laws is the relative orientation of the *a* and *b* axes, which are symmetry equivalent in the tetragonal crystal system and are linked through four-fold rotation along the [001] axis and {100} mirror planes. Furthermore, a non-centrosymmetric space group would only be visible in the reflection intensities, not in their positions. A 120° rotation around one of the <111>_c_ directions would hence produce twins that look identical to 90° twins around the <100>_c_ directions or 180° twins around the <101>_c_ directions. The same also applies to the {011}_c_ reflection twins, that have been reported for this system. Finally, it needs to be noted that the presence of these twin laws does not rule the possibility of further inversion twinning out and, therefore, does not allow a conclusion on the absence of ferroelectricity: Inversion twinning would only be discernible under very specific measurement conditions, for instance at the proximity of an element’s absorption edge, since Friedel’s law is not strictly valid for these cases.

Two structural effects occur during the cubic-to-tetragonal phase transition within the crystal structure of MAPbI_3_: (1) the molecular cations are no longer completely disordered, but orient along a number of preferred orientations and (2) a tilting of the [PbI_6_] octahedra, both of which phenomena are probably interlinked. (1) The molecular cation in the cubic crystal structure is completely disordered and rotates freely in its cuboctahedral void^33^. In the room temperature crystal structure, though, it is still dynamically and statistically disordered but resides in well-defined preferential orientations in correspondence to its surrounding anions^9^. (2) The network of corner-sharing octahedra is undistorted in the cubic aristotype, i.e. all Pb–I–Pb angles are at 180° as dictated by the cubic symmetry. At room temperature, however, the octahedra are still untilted in one direction, i.e. the Pb–I–Pb angles along the crystallographic *c*-axis remain at 180°, but they are tilted within the *ab*-plane. From a crystallographic point of view, the PbI_6_ units no longer form exact octahedra in the tetragonal symmetry, but rather a square-planar bipyramid. The tilting of the inorganic [PbI_6_] octahedra affects the size of the A-cation void (the place of the molecular MA^+^ cation) and on the electronic structure of the compound, which is mainly determined by the inorganic [PbI_6_] octahedra network.

### Neutron Laue diffraction on MAPbI_3_

When applying diffraction methods, one is faced with two distinct problems: on the one hand powder diffraction techniques do not allow to distinguish between *I*4/*mcm* and *I*4*cm* as both belong to the same diffraction group^34^, i.e. they bear exactly the same translation symmetry and hence have the exact same systematic extinctions. Therefore, the only reflections that could differ overlap entirely. Single crystal studies, on the other hand, are flawed by heavy twinning of the crystals under consideration^35^, which is a consequence of the phase behaviour of this compound: MAPbI_3_ shows several structural phase transitions as outlined above.

Applying neutron Laue diffraction to study twinning in methylammonium lead iodide has two major advantages: first, neutrons interact much less strongly with matter than X-rays as neutrons are scattered on the atomic nuclei only, while X-rays are scattered on the electron shell. This implies that larger samples are necessary for neutron diffraction, but also less absorption for the electron rich atoms, such as lead and iodine. The larger size of the samples in neutron diffraction is indeed an advantage for this study, as these crystals typically exhibit more pronounced twinning. Furthermore, the architecture of the instrument is adapted to the use of larger crystals, which is not typically the case for X-ray diffraction with normally much smaller beam sizes of ≈ 500 µm. The chosen setup allowed us to measure crystals of several millimetres size. Furthermore, Laue diffraction has a unique property: by using a polychromatic (“pink”) beam, each individual wavelength constructs its own Ewald sphere and, therefore, allows measurements with static detectors. In a typical single wavelength experiment—which is the standard in X-ray diffraction—the reflections move in and out of the detector plane (i.e. the plane in which the detector records) when the lattice constants change, for instance due to temperature. Therefore, it is generally necessary to measure a crystal during a rotation. In Laue diffraction, on the other hand, the polychromatic light produces reflection spots regardless of lattice parameter changes, and a crystal rotation is not necessary. Using powder diffraction does not allow the distinction of individual domains, since the three-dimensional information of the reflection position is merged into a single dimension, the scattering angle. However, the twinned reflections appear, because more than one domain overlay in a very specific way that is only discernible in three dimensions.

**Figure 3:**
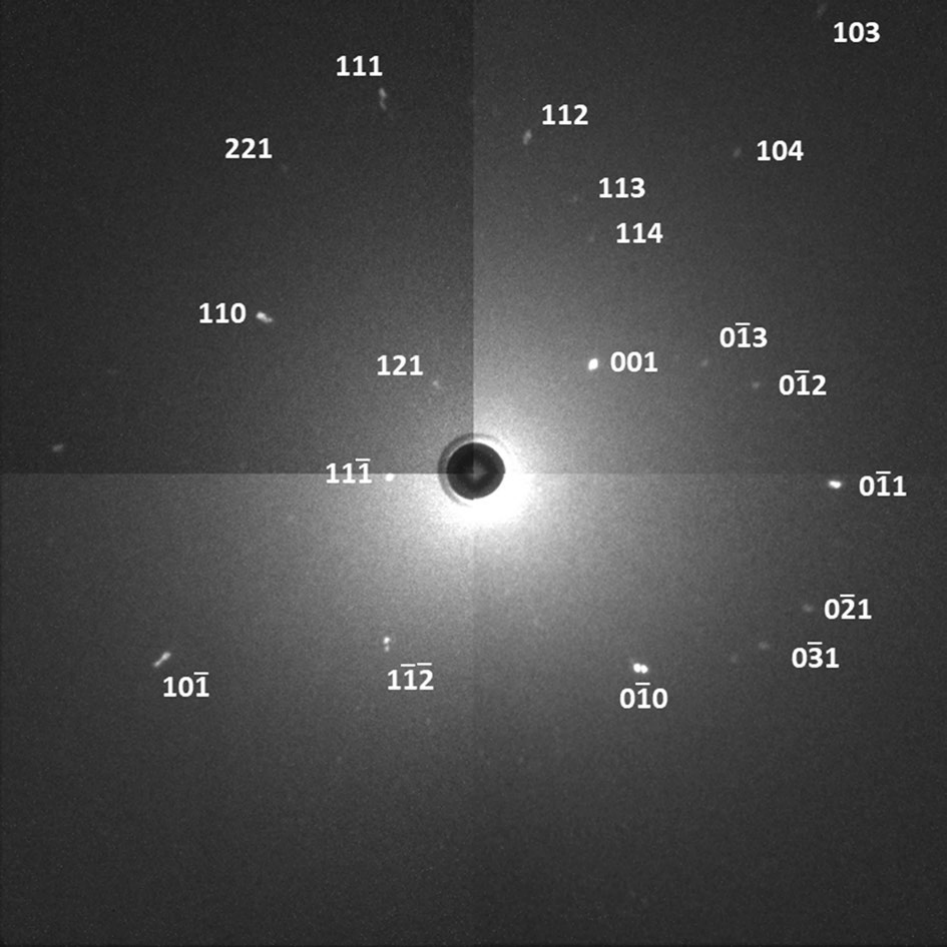
Indexation of a MAPbI_3_ Laue image at room temperature in the cubic aristotype in space group *Pm*$\bar 3$*m* with a = 6.3 Å. The colormap of the image was modified to increase the visibility of the Laue spots. The unaltered Laue image may be found as supplementary information.

Due to the heavy twinning of these crystals, it was impossible to index the neutron Laue patterns using the unit cell parameters of the tetragonal room temperature crystal structure in the space group *I*4*cm* (*I*4/*mcm*) directly. However, indexation was successful on basis of the cubic aristotype, as the differences in the reflection positions between the cubic aristotype and the tetragonal room-temperature structure are rather small. The hkl indices of the cubic indexing are given in Fig. 3, and can be linked to the tetragonal ones through the group-subgroup relationship between the two space groups (comp. Fig. 1). To distinguish the hkl indices in this work, tetragonal body centred, tetragonal face centred and cubic hkls indices are marked with an index t − I, t − F or c, respectively. Furthermore, we checked the successful indexation using the tetragonal subgroup *P*4/*mmm* (see “SI”), which is an intermediate space group in the symmetry descent but still bears approximately the same lattice constants as the cubic aristotype. In essence all three axis permutations appear as equally acceptable solutions for the indexation.

**Figure 4:**
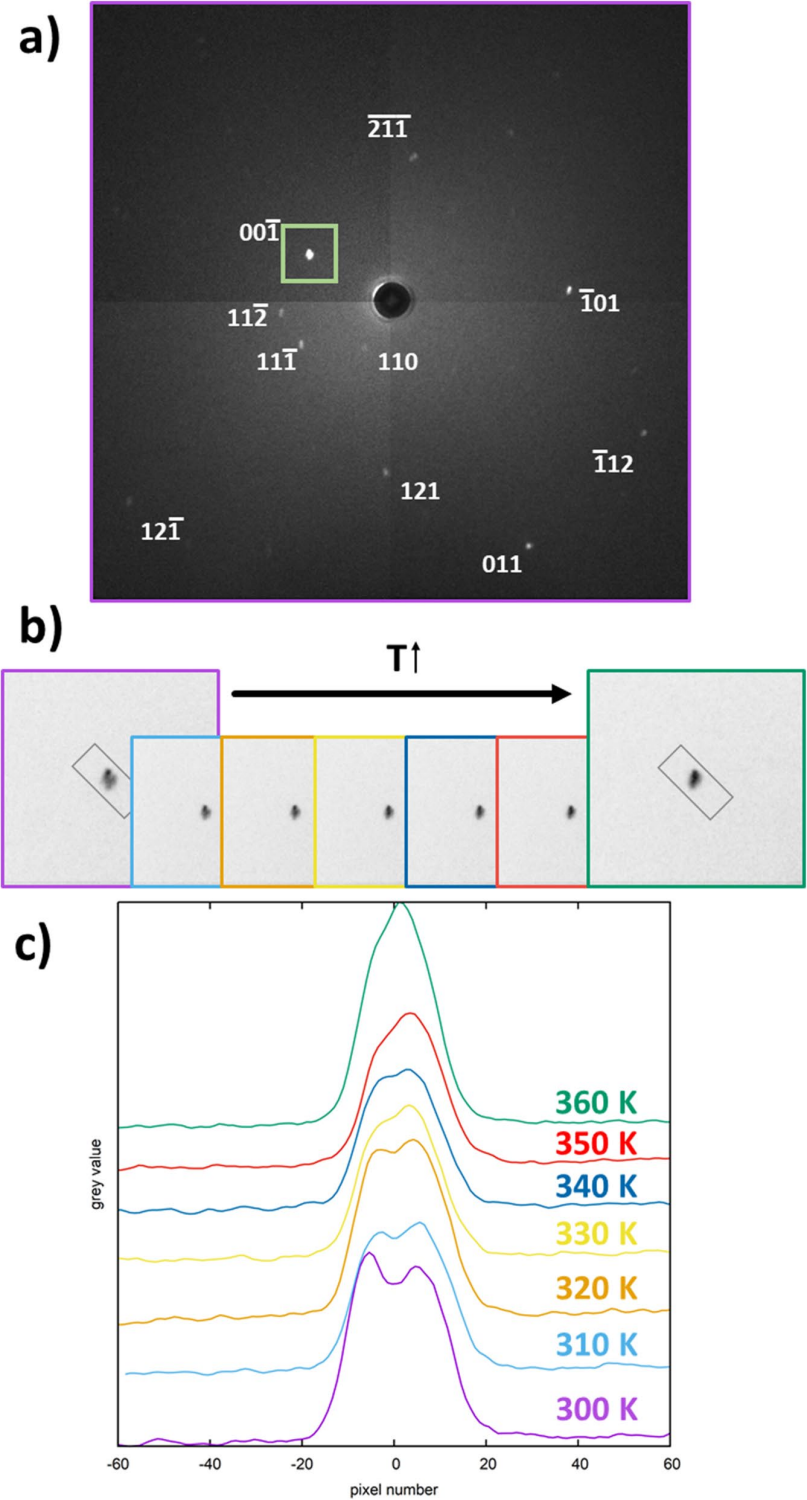
Indexation of the transmission Laue image of MAPbI_3_ at 300 K in the cubic aristotype (**a**). The colormap was modified to increase spot visibility. The unaltered Laue image may be found as supplementary information. The inverted image of the area selected around the 00$\bar 1$_c_ reflection (grey frame in **a**) is shown at variable temperature (**b**). Rectangular profile integration at different temperatures (**c**) was performed for the selected area shown in **b**).

Using a static crystal in a special sample environment, we conducted temperature-dependent measurements using a specific crystal orientation. Due to the sample environment, we were limited in the choice of orientation, which is why the orientations at room temperature and at controlled temperature differ. The Laue patterns clearly show a twinning at room temperature in a pseudo-merohedral way: the double-maxima of the spots under consideration lie very close to each other (Fig. 4a). However, the Laue spots are clearly split at 300 K (Fig. 4b,c). On heating towards the tetragonal-to-cubic phase transition temperature at 330 K, the spots merge more and more into one single spot as the reflections of the twins become equivalent and hence the twinning domains are being merged during this process. The smooth merging of the domains can be taken as an indicator for the fact that this phase transition is actually an order–disorder phase transition.

**Figure 5:**
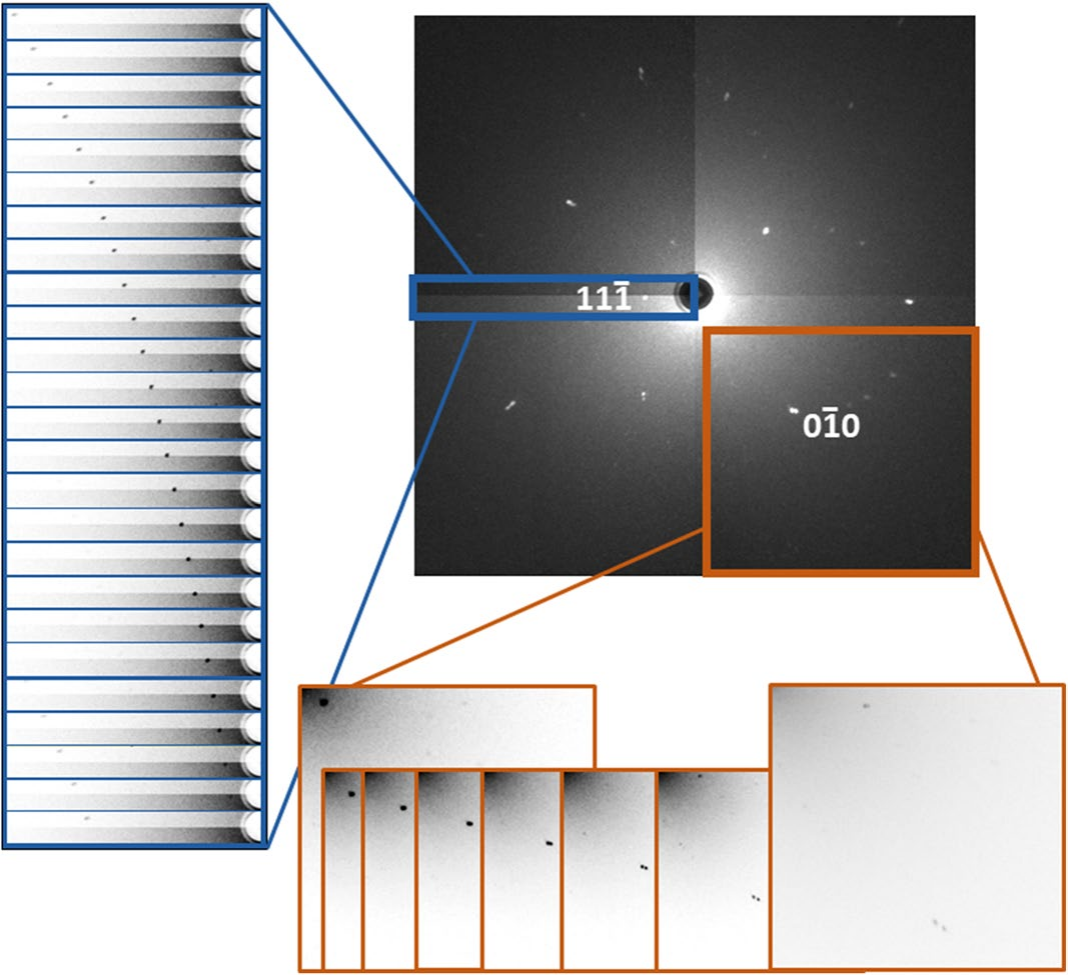
Changes of the 0$\bar 1$0_c_ spots during an ω-scan (5° steps) and of the 11$\bar 1$_c_ spots during the same scan (1° steps). The cut-outs were inverted and brightness/contrast adjusted to increase visibility of the spots.

In order to prove the nature of the twinning at room temperature, we recorded a series of diffraction images while turning the crystal in increments of 1° around the ω-axis (i.e. the axis, on which the crystal is mounted and that is perpendicular to the beam path) without the heating equipment. What is observed in such an experiment is the movement of the diffraction spots through the detector plane, caused by diffraction at the same lattice plane from different wavelengths at different crystal rotations. According to Bragg’s law, the separation of reflections depends on the wavelength (as a simplification, the wavelength of the twinned reflections with very similar *d*-spacings shall be taken as equal) and becomes larger at longer wavelengths, since *d* and λ are proportional to each other. It can be observed that most reflections show a separation indicative of twinning, since they bear two reflections that are almost, but not completely, identical in *d*-spacing. As an example, this behaviour can be very well observed for the 110_t−I_ (200_t−F_ and 020_t−F_) and 002_t−I_ (002_t−F_) reflections (equivalent to the 0$\bar 1$0_c_ reflections, Fig. 5). However, the 11$\bar 1$_c_ reflection behaves differently in that no splitting can be observed. Since all the lattice planes {111}_c_ still exhibit the same *d*-spacing in the tetragonal phase ({202}_t−I_ or {222}_t−F_), they are diffracted by the same wavelength and hence do not vary in spot separation in the detector plane. Consequently, the 11$\bar 1$_c_ reflection does not exhibit a change in splitting over its full way through the detector plane, in line with rotation twinning along the three-fold <111> axes. As we grew the crystals used in this experiment at room temperature, the twinning clearly does not stem from running through the cubic-to-tetragonal phase transition. Instead, a different symmetry between the crystallisation nuclei and the bulk material may be an explanation for this behaviour, where the nuclei have a higher symmetry than the bulk material.

## Conclusion

We demonstrated the particularities of twinning in MAPbI_3_ by means of neutron Laue diffraction and explained the peculiarities of twinning in MAPbI_3_ from a crystallographic point-of-view. We could show intrinsic twinning in crystals grown at room temperature, which have not been heated to the existence region of the cubic phase prior to the experiments. This latter gives a powerful insight into the crystallisation and growth process of MAPbI_3_ and supports the assumption of cubic crystallisation nuclei. This finding is important as it may allow more targeted thin film production procedures in the future, and more targeted studies on the crystallographic nature of the earlier crystallisation states will be highly beneficial.

## Materials and methods

Single crystals for this study were grown using an antisolvent room temperature method described before^16,21^. Crystals were covered in acrylic lacquer to prevent oxygen and moisture destruction of the crystal.

Neutron Laue patterns were collected using the FALCON (Fast Acquisition Laue Camera for Neutrons) Laue diffractometer at BER II research reactor (HZB). FALCON consists of two area detectors in backscattering and transmission position with four iCDD cameras each and total scattering area of 400 × 400 mm^2^. A “pink” neutron beam with wavelength $\approx$ 0.9–3.2 Å and a neutron flux of 8 × 10^6^ n/cm^2^s is applied. For variable temperature measurements, a detector distance for the Laue patterns was 168 mm, pattern acquisition time was 60 s. Temperature depending measurements were carried out by means of a Stirling cooler based closed cycle cryostat providing sample temperatures from 150 up to 450 K^36^. Room temperature measurements were performed at a detector distance of 120 mm with an acquisition time of 180 s. By means of a rotation stage, ω-scans were performed within the range of − 120° and 120°. For indexing of the Laue patterns “Cologne Laue Indexation programme” CLIP^37^ was applied. Successful indexation was possible assuming perovskite cubic aristotype with a unit cell dimension of *a* = 6.3 Å. Further, indexation was performed in the tetragonal subgroup *P*4/*mmm*, as this subgroup still bears approximately the same unit cell dimensions of the cubic space group (see “SI”), but clearly shows the twinning through axis permutation.

## Supplementary information

Supplementary Information.
