# Supplementary material for: Twinning in MAPbI3 at room temperature uncovered through Laue neutron diffraction
Source: Sci Rep. 2020 Oct 6;10:16613. doi: 10.1038/s41598-020-73487-1 (PMC7538425; doi:10.1038/s41598-020-73487-1)

Supplementary Information for

**Twinning in MAPbI_3_ at room temperature uncovered through Laue neutron diffraction**

Joachim Breternitz,^a^ Michael Tovar,^a^ Susan Schorr^a,b^

a Helmholtz-Zentrum Berlin für Materialien und Energie GmbH, Department Structure and Dynamics of Energy Materials, Hahn-Meitner-Platz 1, 14109 Berlin, Germany.
b Freie Universität Berlin, Department Geosciences, Malteserstr. 74-100, 12249 Berlin, Germany.

Original Laue images


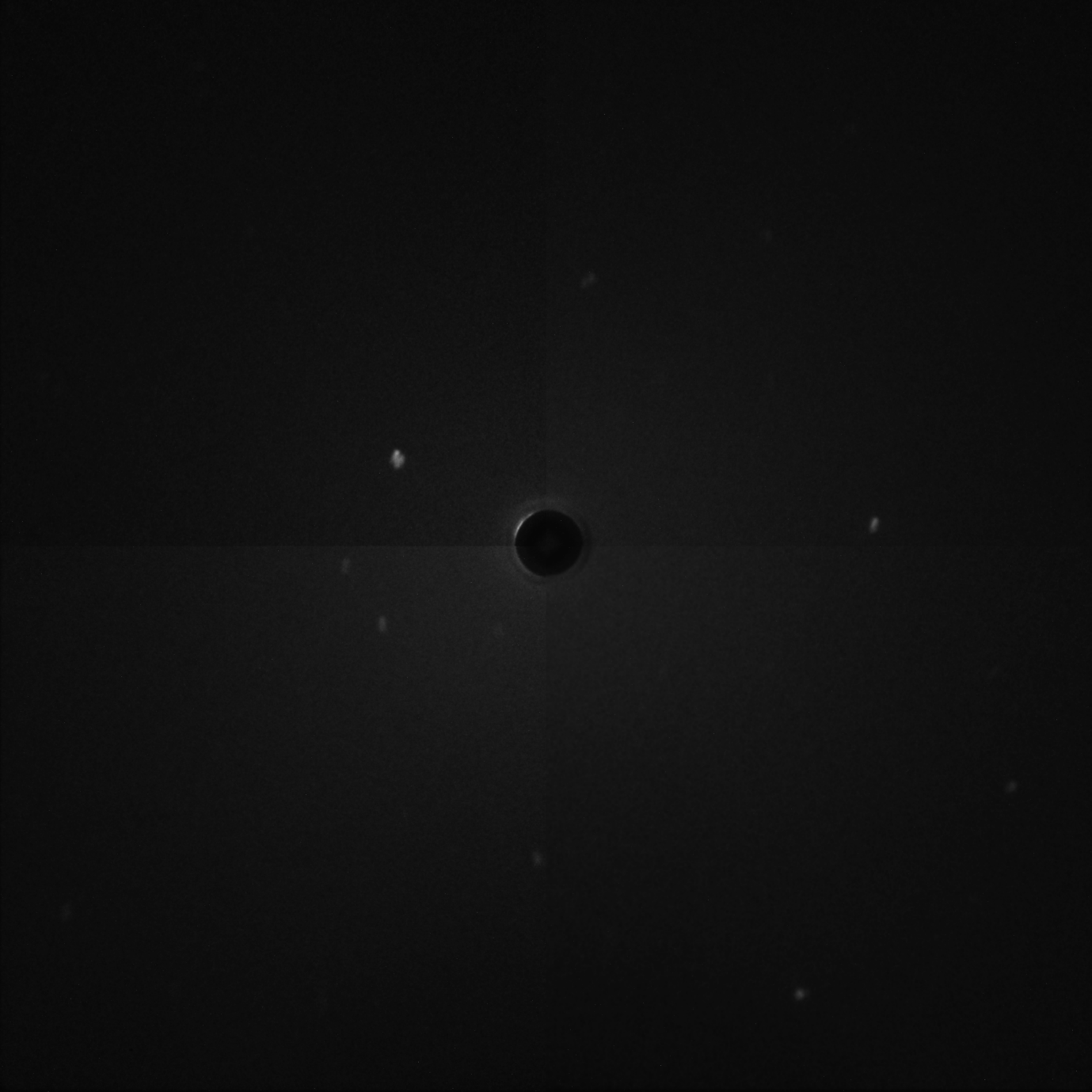


FigureS1: Original Laue image at 300 K in the temperature run, as shown in Figure 2.


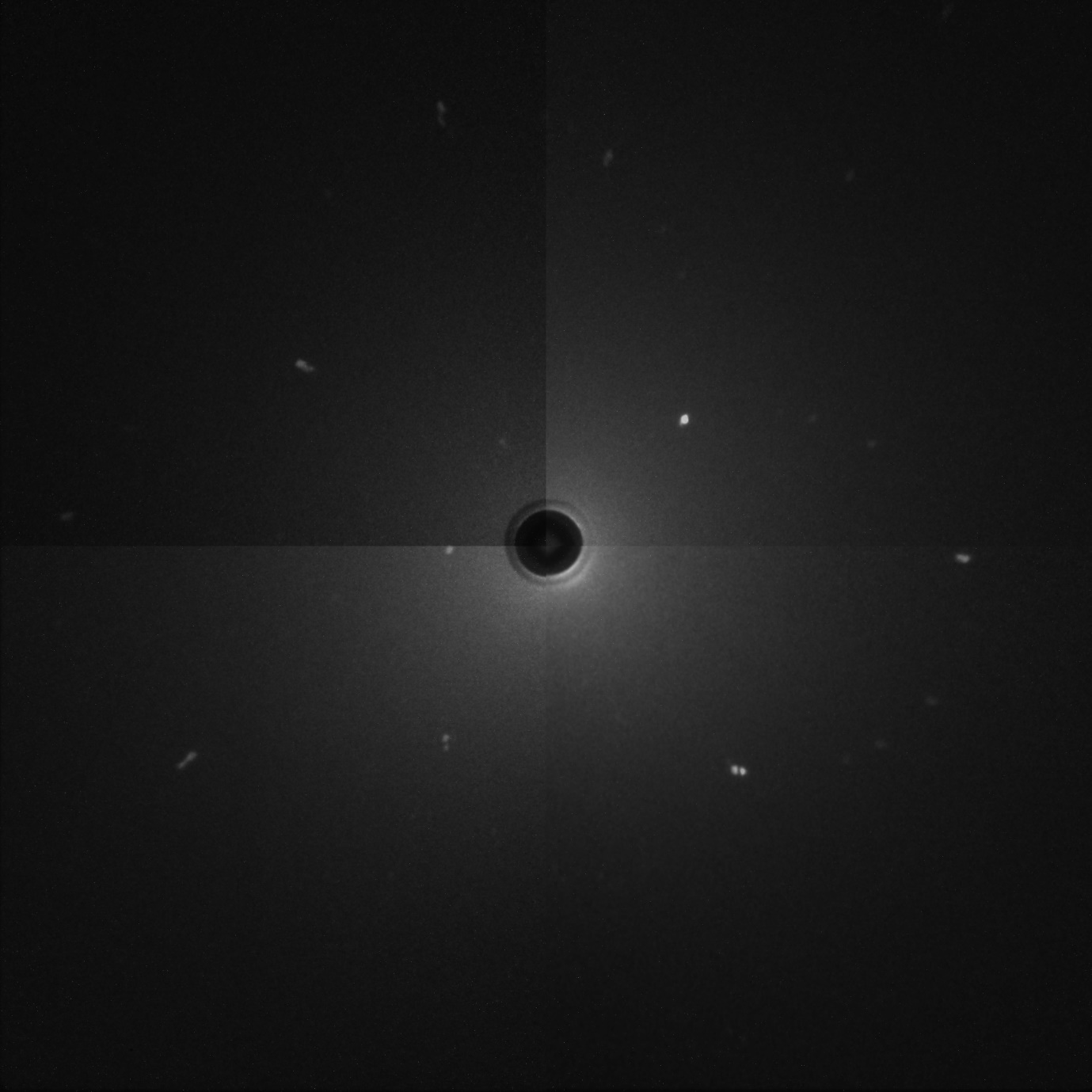


Figure S2: Unaltered Laue image in the omega-scan, as displayed in figures 3 and 4.

**Indexation of the pattern in the tetragonal space group *P*4/*mmm***

The six first indexation solutions sorted by deviation in the following figures all have very similar deviation parameters and represent axis permutations of the tetragonal unit cells. For convenience, we show the indexations as they are produced by the program to demonstrate the similarities of these solutions.


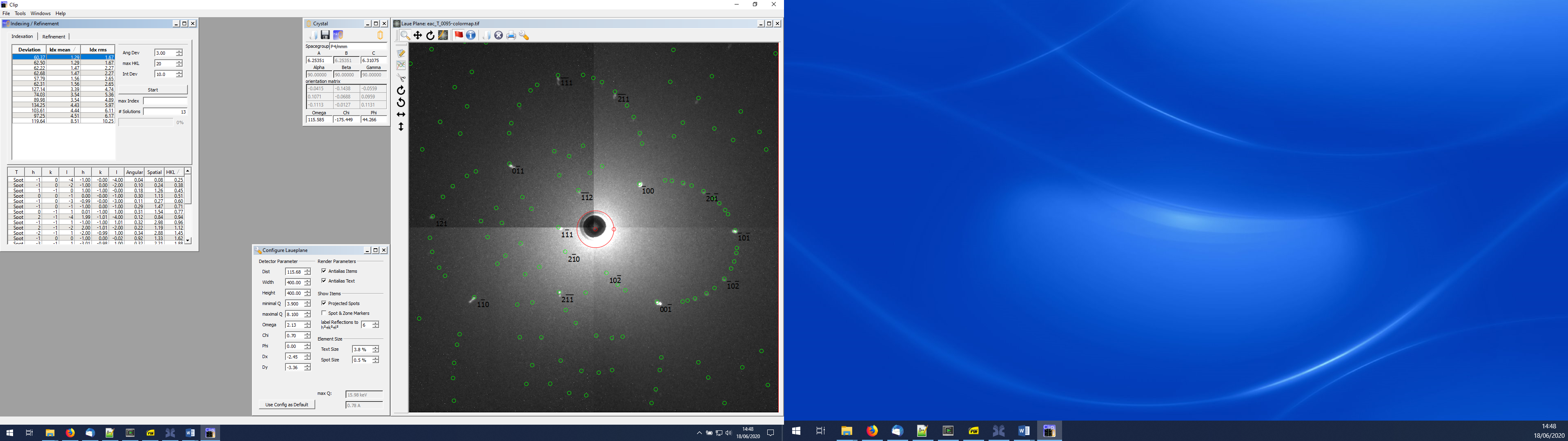


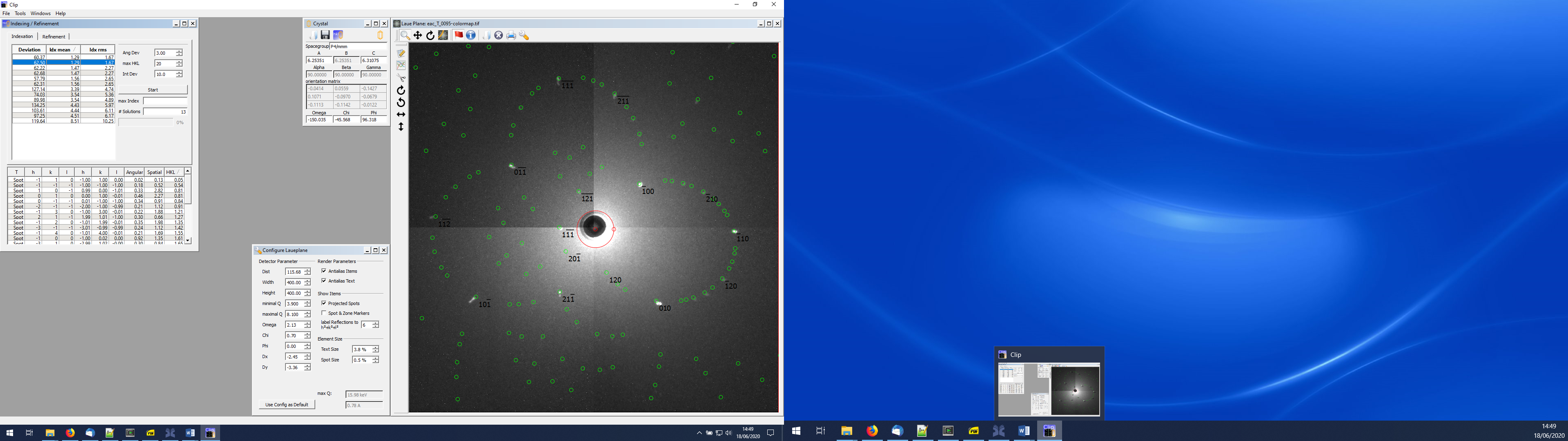


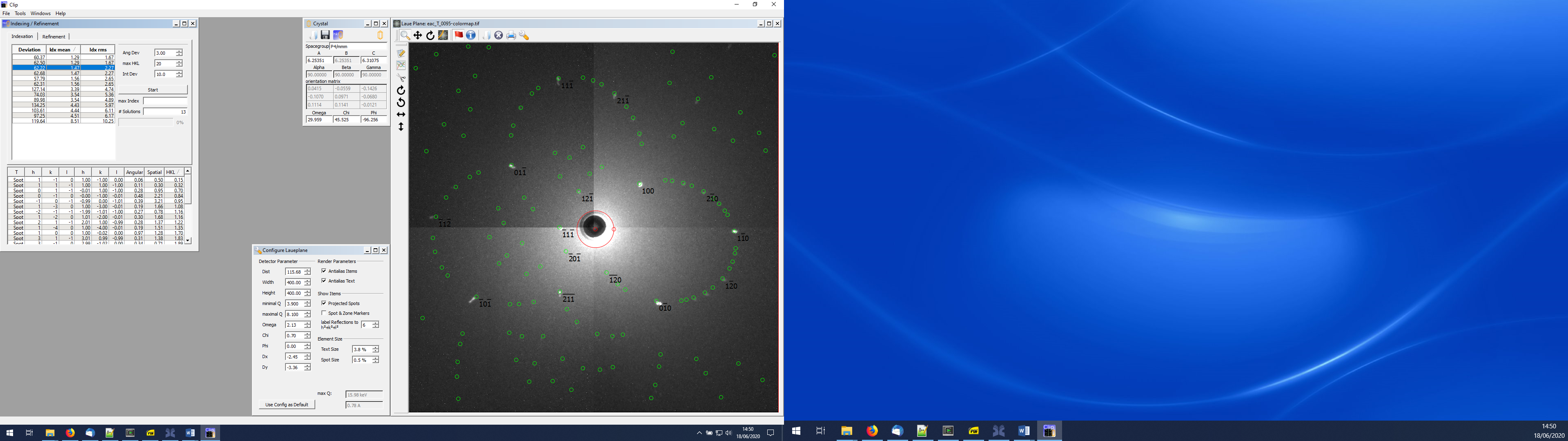


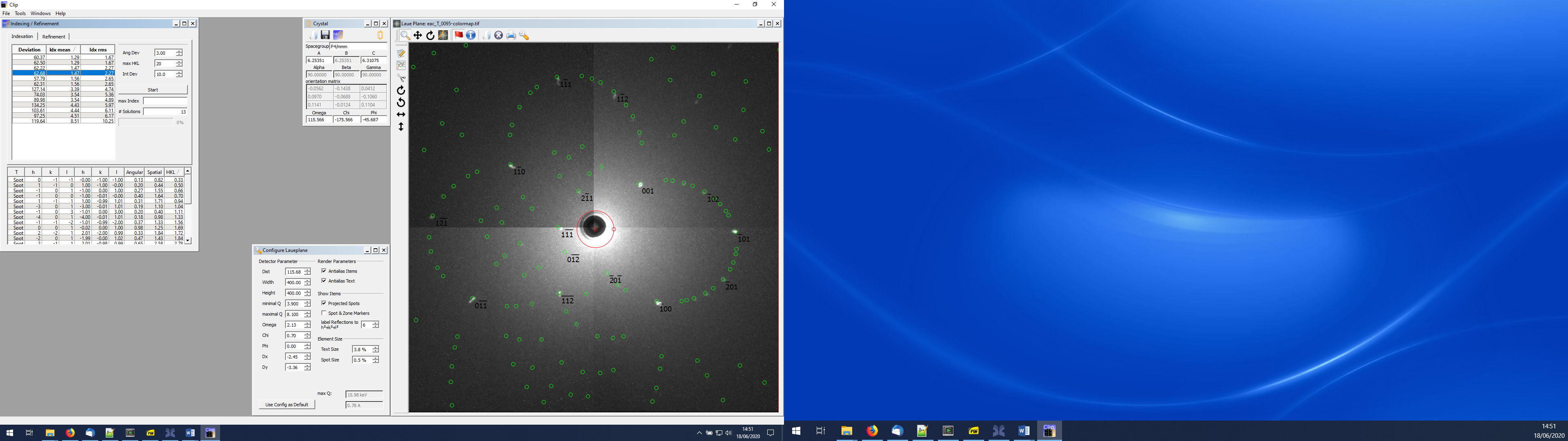


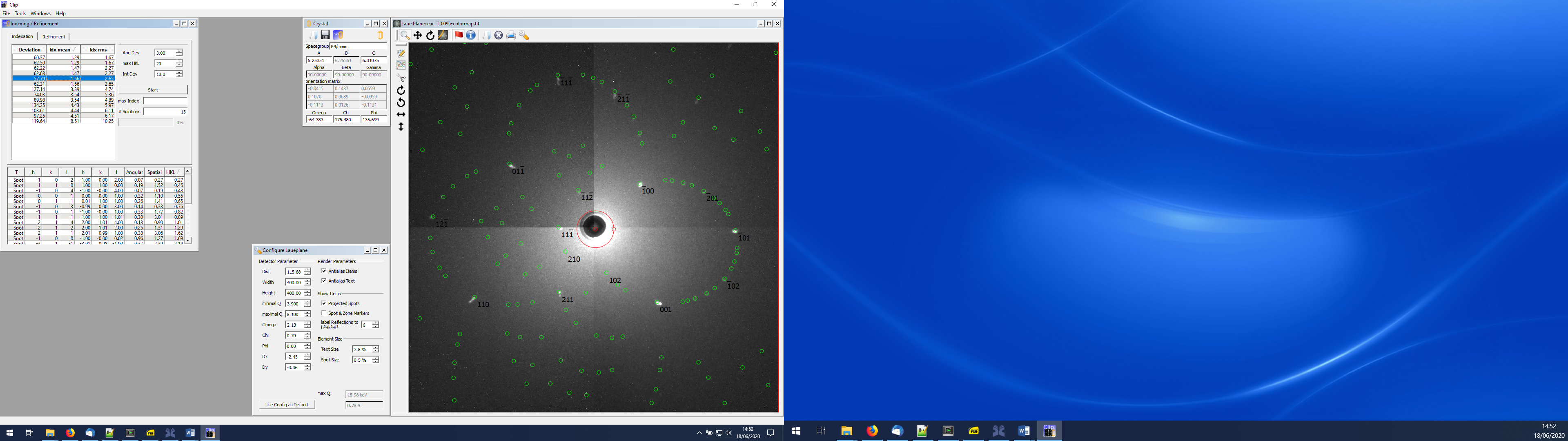


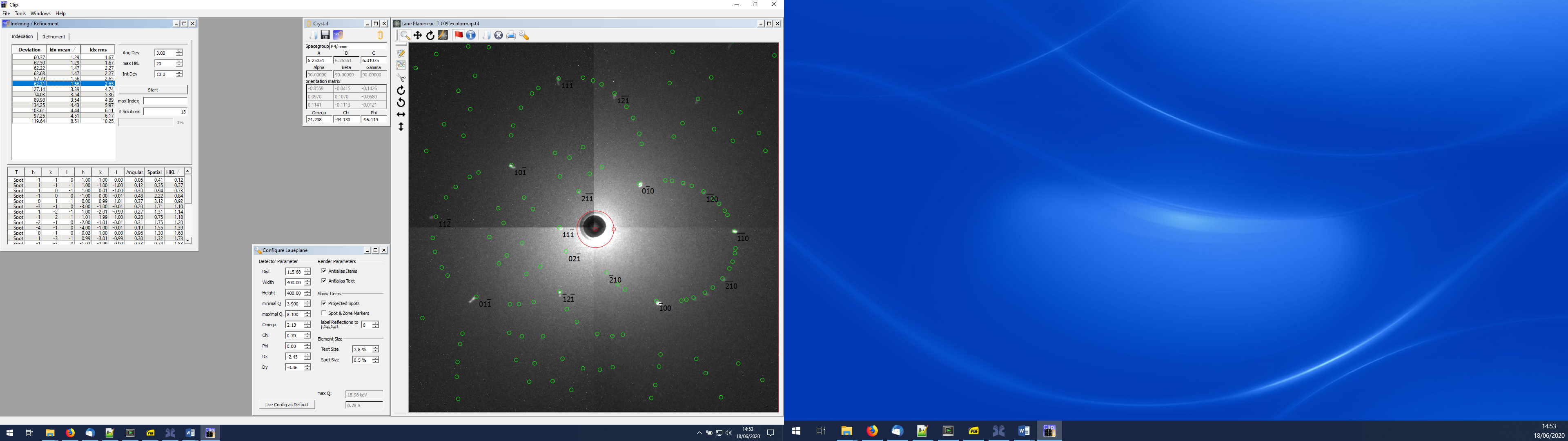

Supplement: Supplementary file 1 — Supplementary Information. [file 41598_2020_73487_MOESM1_ESM.docx]
